# Coherence vortices by binary pinholes

**DOI:** 10.1515/nanoph-2024-0380

**Published:** 2024-10-17

**Authors:** Akanksha Gautam, Amit K. Agarwal, Rakesh Kumar Singh

**Affiliations:** Laboratory of Information Photonics and Optical Metrology, Department of Physics, Indian Institute of Technology (Banaras Hindu University), Varanasi 221005, India; Photonics Division, Instruments R&D Establishment, Dehradun 248001, India

**Keywords:** coherence vortices, topological charge, orbital angular momentum, spatial coherence

## Abstract

Singularity in a two-point complex coherence function, known as coherence vortices, represents zero visibility with a helical phase structure. In this paper, we introduce a novel technique to generate the coherence vortices of different topological charges by incoherent source transmittance with exotic structured binary pinholes. The binary pinhole structures have been realized by lithography, followed by wet etching methods. We control the transmittance from the incoherent source plane using these exotic apertures, which finally results in a coherence vortex spectrum that features multiple and pure orbital angular momentum modes. The generation of the coherence vortices is achieved within the two-point complex spatial coherence function. The spatial coherence function exhibits the helical phase profile in its phase part, and its absolute part shows a doughnut-shaped structure. A theoretical basis is developed and validated with simulation, and experimental results. The coherence vortex spectra with OAM modes superposed with opposite topological charges, known as photonic gears, are also generated with the proposed theory.

## 1 Introduction

Singular optics, an area of intense research interest, addresses a diverse range of effects that occur close to locations where certain parameters of the wave field become singular or undefined [1], [2], [3], [4], [5], [6], [7], [8]. Over the last few decades, researchers have described several different kinds of singularities in the optical domain, such as phase singularities [1], [2], [3], [4], [5], polarization singularities [6], [7], and Poynting vector singularities [8]. Among all, phase singularities have attracted significant attention because of their capacity to carry orbital angular momentum (OAM) [9] and their various useful applications, including free-space optical communication [10], particle trapping and manipulation [11], [12], quantum computing [13], super-resolution imaging [14], and many more.

The vortex beams (VBs) exhibit intensity null around the phase singularity and the phase is undefined [4]. These beams are characterized by a helical phase wavefront with azimuthal phase dependence in the form of exp(*ilθ*), where *l* is the topological charge (TC) and *θ* is the azimuthal phase over the transverse plane. The total phase change around the phase singularity is 2*πl*, and the TC *l* is associated with the OAM of the photon as l*ℏ*, where *ℏ* denotes the Planck constant [15]. Over the past few decades, various methods for generating phase singularities have been introduced. These include spiral phase plates [16], computer-generated holograms [17], light mode converters [18], plasmonic metasurfaces [19], and so on. Recently, fully coherent light arrays designed in a spiral structure have been used to create an optical vortex with a pure or multiple OAM. The specifically designed spiral structure provides freedom to tailor OAM spectrum of the beam [20], [21], [22]. Also, numerous techniques have been devised to identify the TC of a VB. Shack–Hartmann wavefront sensors [23], diffraction techniques [24], [25], interferometry [26], scattering [27], diffraction by pinhole [28], and ptychography [29] are a few of them.

The aforementioned research is mainly limited to coherent beams. Nonetheless, a coherent beam will distort, scintillate, or drift when it passes through a turbulent medium like fog or heat movement. However, a low coherent or partially coherent beams (PCB) have advantages over fully coherent beams in mitigating turbulence-induced effects [30]. Additionally, PCBs with vortex exhibit even greater advantages compared to those without vortex, particularly in minimizing turbulence-induced scintillations, presenting significant potential benefits for free-space optical communication [31], [32]. The partially coherent vortex beam (PCVB) is also used in optical imaging domains to minimize speckle noise [33] and in trapping particles having varying refractive indices [34]. Moreover, certain PCVB shows some special properties while propagation, such as self-splitting, self-focusing, and self-reconstruction making it suitable for applications such as information encryption and decryption [35], [36]. As a PCVB propagates, the phase singularities corresponding to the central zero intensity diminish, while correlation singularities characterized by zero cross-spectral density (CSD) function and undefined phase arise. Therefore, for PCV beams, while the random fluctuations of the light fields obscure the phase singular points of the averaged intensity, the singularities survive in the two-point correlation as correlation singularities or coherence vortices (CVs) [37]. Gbur and colleagues were the first to theoretically predict the presence of CVs. CVs contain a helical phase structure within a two-point correlation function [38]. The existence of CVs as ring dislocations in the cross-correlation function was first realized by Palacios et al. [39]. Subsequently, Wang et al. showed the existence of CVs in their generic form, exhibiting characteristic helical phase profiles associated with complex spatial coherence function [40]. Additionally, the method is applied to establish the law of conservation and to examine the local properties of the phase singularities in the spatial coherence function [41]. Singh et al. proposed a method involving three uniform independent circular apertures at the source plane to generate a coherence vortex array [42]. Recently, Liu et al. showed an experimental generation of coherence vortex by utilizing partially coherent light arrays [43]. Therefore, owing to the numerous benefits and advantages of CVs, it is essential to generate and detect CVs.

The detection of CVs serves a crucial purpose in measuring the TC, which proves highly beneficial in applications such as information photonics, and optical communication [44], [45]. Over the past decade, different techniques have been devised for measuring TCs using a variety of correlation functions, including the double-correlation function [46], complex degree of coherence [47], and cross-spectral density [48]. Subsequent studies [47], [49] revealed a direct connection between the number of ring dislocations observed in the cross-correlation function and the magnitude of the TC associated with a PCVB [50]. Theoretical investigations [51], [52] have further elucidated the relationship between the CVs and the magnitude of the TC, as well as the radial mode index of the vortex beam [53]. A technique utilizing dual cylindrical lenses for simultaneously measuring the magnitude and sign of the TC was proposed [54]. The determination of the TC of CVs has advanced through recent developments in phase detection of PCVB [48], [55], [56]. Recently, a phase detection method is proposed to enable simultaneous determination of both the amplitude and sign of the TC [56].

However, the generation of CVs with arbitrary TC composition is still unexplored except for some limited studies with a fixed TC. The purpose of this paper is to fill this gap and demonstrate an experimental method to generate and analyze the CV spectrum with pure and multiple OAM modes. For this purpose, we have specifically fabricated binary pinhole masks using lithography. Apart from a synthesis of the CVs, we have also designed a highly stable interferometer to experimentally measure the two-dimensional complex coherence function and confirm the presence of the CVs in two-point spatial coherence function at different longitudinal distances from the source plane. With this approach, using a single setup, we can retrieve the characteristic doughnut structure and helical phase profile of CVs in amplitude and phase part of complex two-point spatial coherence function flow in the three-dimension, respectively. Also, employing the same setup we generate the photonic gears in the coherence, *i.e.*, OAM modes superposition with opposite TCs ± *l*. We have developed a theoretical framework and validated it with simulations and experimental tests. Additionally, the compositions of the topological spectrum in the CVs are confirmed by projecting the recovered complex field over the helical modes, and results are presented. The idea presented here is not limited to optical regime and this kind of pinhole mask can be further investigated for matter waves, e.g., in the context of photon sieves and Vogel spiral arrays, enabling the creation of more intricate structures and a greater diversity of structured beams [57], [58], [59], [60].

## 2 Principle and methods

### 2.1 Generation of coherence vortex with varying modes

The CSD function describes the spatial correlations of the field. For a two-dimensional wide-sense stationary source, it is represented as

(1) $$W\left(r_{1},r_{2};\omega \right)=\left\langle E^{*}\left(r_{1};\omega \right)E\left(r_{2};\omega \right)\right\rangle ,$$

where, $E\left(r_{1};\omega \right)$ and $E\left(r_{2};\omega \right)$ represents the far-field realizations at spatial points ***r***_1_ and ***r***_2_, respectively, and *ω* is the frequency. 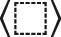 represents ensemble averaging.

**Figure 1:**
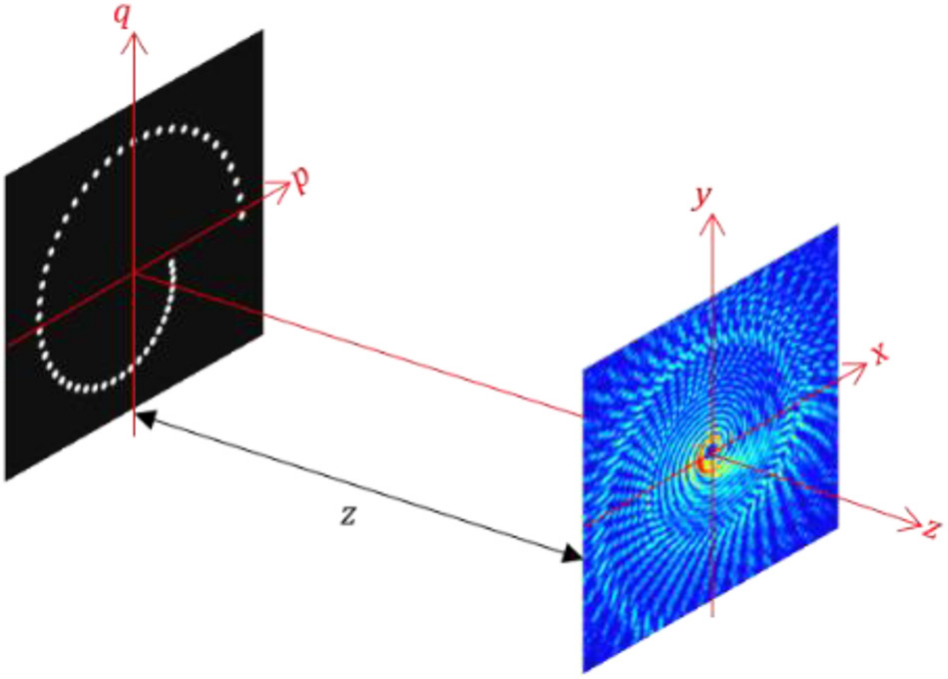
Schematic diagram for recording two-point spatial coherence function.

Figure 1 shows a binary spiral aperture illuminated with an incoherent light, using the angular spectrum of plane waves, the complex field at a distance *z* from the aperture plane can be expressed as [61]

(2) $$E\left(r;\omega \right)=\iint a\left(p,q;\omega \right)\exp\left[i\left(px+qy+mz\right)\right]dp\text{d}q,$$

where $\left(x,y,z\right)\equiv \left(r,z\right)$ , ***r*** is the transverse position vector at a longitudinal plane *z*, $m=\sqrt{k^{2}-p^{2}-q^{2}}$ and $k=\frac{2\pi }{\lambda }$ denotes wave number, *λ* is the wavelength and $a\left(p,q;\omega \right)$ is the amplitude of the field. We neglected the contributions of the evanescent waves for a beam-like field.

On substituting Eq. (2) into Eq. (1), we obtain

(3) $$\begin{array}{ll} & W\left(r_{1},r_{2},z_{1},z_{2};\omega \right)\\ & =∭\;\int A\left(p_{1},q_{1},p_{2},q_{2};\omega \right)\exp\left[i\left(p_{2}x_{2}+q_{2}y_{2}+m_{2}z_{2}\right.\right.\\ & \;\left.\left.-p_{1}x_{1}-q_{1}y_{1}-m_{1}z_{1}\right)\right]\text{d}p_{1}\text{d}q_{1}\text{d}p_{2}\text{d}q_{2}, \end{array}$$

where $A\left(p_{1},q_{1},p_{2},q_{2};\omega \right)=\left\langle a^{*}\left(p_{1},q_{1};\omega \right)a\left(p_{2},q_{2};\omega \right)\right\rangle$ , represents the angular correlation function of the field. If *p* ^2^ + *q* ^2^ ≪ *k* ^2^,

(4) $$m\approx k\left[1-\left(\frac{p^{2}+q^{2}}{2k^{2}}\right)\right].$$

Therefore, Eq. (3) becomes

(5) $$\begin{array}{ll} & W\left(r_{1},r_{2},z_{1},z_{2};\omega \right)\\ & =\exp\left[ik\left(z_{2}-z_{1}\right)\right]∭\;\int A\left(p_{1},q_{1},p_{2},q_{2};\omega \right)\exp\left[i\left(p_{2}x_{2}\right.\right.\\ & \;\left.\left.+q_{2}y_{2}-p_{1}x_{1}-q_{1}y_{1}\right)\right]\exp\left\{\frac{i}{2k}\left[\left(p_{1}^{2}+q_{1}^{2}\right)z_{1}\right.\right.\\ & \;\left.\left.-\left(p_{2}^{2}+q_{2}^{2}\right)z_{2}\right]\right\}\text{d}p_{1}\text{d}q_{1}\text{d}p_{2}\text{d}q_{2}. \end{array}$$

Considering $v_{1}\equiv \left(p_{1},q_{1}\right)$ and $v_{2}\equiv \left(p_{2},q_{2}\right)$ be the two-dimensional vectors at the transverse source plane. Thus, Eq. (5) reduces to

(6) $$\begin{array}{ll} & W\left(r_{1},r_{2},z_{1},z_{2};\omega \right)\\ & =\exp\left[ik\left(z_{2}-z_{1}\right)\right]\iint A\left(v_{1},v_{2};\omega \right)\exp\left[i\left(v_{2}\cdot r_{2}-v_{1}\cdot r_{1}\right)\right]\\ & \;\times \exp\left[-\frac{i\left({\left|v_{2}\right|}^{2}z_{2}-{\left|v_{1}\right|}^{2}z_{1}\right)}{2k}\right]{\text{d}}^{2}v_{1}{\text{d}}^{2}v_{2}, \end{array}$$

where for an incoherent source $A\left(v_{1},v_{2};\omega \right)=\left\langle a^{*}\left(v_{1};\omega \right)a\left(v_{2};\omega \right)\right\rangle =I\left(v;\omega \right)\delta \left(v_{1}-v_{2}\right)$ . Therefore, Eq. (6) becomes

(7) $$\begin{array}{ll} W\left(\Delta r,\Delta z;\omega \right) & =\exp\left(ik\Delta z\right)\int I\left(v;\omega \right)\\ & \;\times \exp\left(iv\cdot \Delta r\right)\exp\left[-\frac{i}{2k}{\left|v\right|}^{2}\Delta z\right]{\text{d}}^{2}v, \end{array}$$

where Δ*z* = *z*_2_ − *z*_1_ and Δ ***r*** = ***r***_2_ − ***r***_1_. When Δ*z* = 0, Eq. (7) reduces to the van Cittert–Zernike theorem which connects the two-point spatial coherence function with the incoherent source by the Fourier transform relation. Therefore, for Δ*z* = 0,

(8) $$W\left(\Delta r;\omega \right)=\int I\left(v;\omega \right)\exp\left(iv\cdot \Delta r\right){\text{d}}^{2}v,$$

where, $I\left(v\right)$ is a real, non-negative weight function. Therefore, defining function $I\left(v\right)$ with an appropriate spatial structure is sufficient to produce a specified far-field spatial coherence distribution. For a monochromatic light source, we have ignored *ω* from further consideration in the coming section.

Following beam shaping of the coherent light [21] and using the analogy between the optical field and complex coherence, we create an exotic incoherent source with structured transmittance $I\left(v\right)$ . This is made up of several pinholes positioned at different spatial locations in the form of pinholes arranged in a spiral structure as shown in Figure 1. Experimental fabrication of the desired transmittance function for high-quality performance is discussed in the next section. The transmittance aperture is made up of the total *N* number of pinholes, the radius $v\left(n\right)$ and azimuthal angle $\theta \left(n\right)$ of *n*th pinhole from the center is set over the transverse plane such that

(9) $$v\left(n\right)={\left(\frac{lz\lambda \theta \left(n\right)}{\pi }+v_{0}^{2}\right)}^{\frac{1}{2}},$$

and

(10) $$\theta \left(n\right)=\frac{2\pi n}{N},$$

where, *v*_0_ is the initial radius from the center to the first pinhole, *l* is the TC, *λ* is the wavelength and *z* is the distance from the pinhole mask to the observation plane.

To examine the generation of CVs, we implemented a two-pronged approach. First is the measurement of complex coherence. The presence of helical phase structure in order of multiple of 2*π* is used to test and confirm the CV in the beam. Second is an analysis based on examining the composition of the TCs spectrum in the beam. The orthogonal projection method is implemented to analyze the TCs spectrum in the low coherent beam. Here, the complex coherence function $W\left(\Delta r\right)$ is projected onto spiral harmonics $\exp\left(il\phi \right)$ , where *l* represents the TC [62], [63]. To examine the TC power spectrum, we determine the complex coefficient *A*_*l*_ by applying an angular Fourier transform of $W\left(\Delta r\right)$ over azimuthal angle,

(11) $$A_{l}\left(\Delta r\right)=\frac{1}{2\pi }\overset{2\pi }{\underset{0}{\int }}d\phi \exp\left(-il\phi \right)W\left(\Delta r\right).$$

Every TC mode is associated with a complex coefficient *A*_*l*_, that varies with the radial coordinates. The TC mode power spectrum of the beam is then calculated using a numerical integration across the modulus square of *A*_*l*_ for the radial coordinates,

(12) $$P\left(l\right)=\frac{1}{S}\overset{\infty }{\underset{0}{\int }}dr\;r{\left|A_{l}\left(\Delta r\right)\right|}^{2},$$

where, $P\left(l\right)$ represents the TCs power spectrum and $S=\sum \overset{\infty }{\underset{0}{\int }}dr\;r{\left|A_{l}\left(\Delta r\right)\right|}^{2}$ is the beam power.

**Figure 2:**
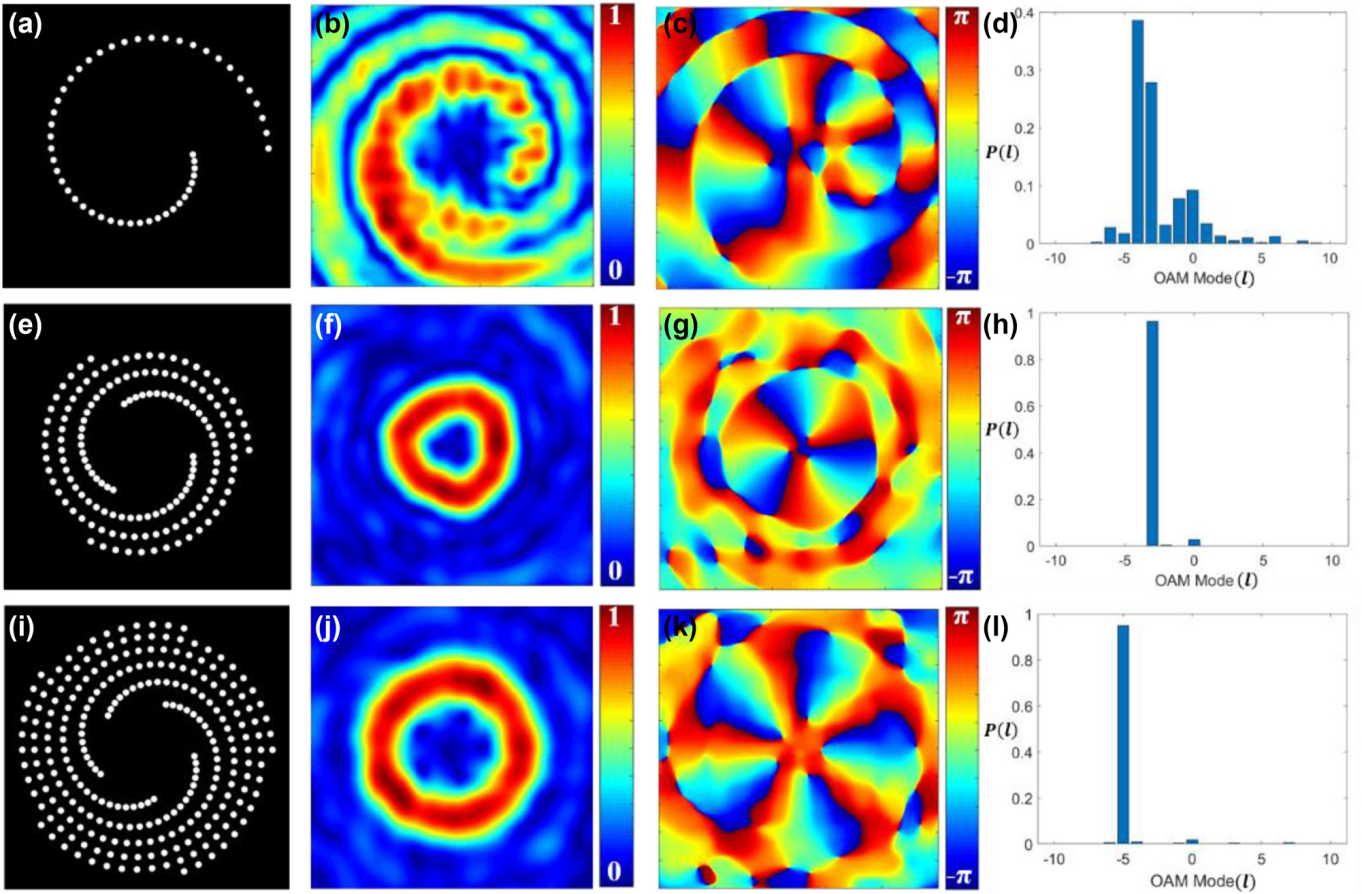
Schematics of the discretized binary spiral pinhole apertures. (a) Single spiral, (e) three spirals, and (i) five spirals; (b), (f), and (j) the corresponding simulated absolute values of complex spatial coherence function; (c), (g), and (k) the corresponding simulated phase values of complex spatial coherence function; (d), (h), and (l) the corresponding TC mode power spectrum.

To examine the proposed method, we have simulated the incoherent source with binary pinholes and demonstrated generation of CVs in the beam. For simulation, we modeled the spiral pinhole aperture $I\left(v\right)$ using Eqs. (9) and (10). The source plane is comprised of the pinhole aperture expressed as a two-dimensional matrix $I\left(a,b\right)$ , with *L* × *L* elements, where *a* and *b* are pixels. An incoherent source is modeled using a set of random phase screens. These phase screens introduce random phase values uniformly distributed over the interval [–π, π]. Propagation of incoherent beam through the transmittance function is modeled using angular spectrum method and complex coherence is further propagated using Eq. (7). In simulation, we considered the initial radius from the center to the first pinhole to be *r*_0_ = 1 mm, wavelength *λ* = 632.8 nm, total number of pinholes *N* = 50, Δ*z* = 1 m. Figure 2 displays our simulated results.

Figure 2(a) shows the binary spiral pinhole aperture. Figure 2(b) and (c) display the amplitude and phase parts of the simulated complex spatial coherence function arising from incoherently illuminated spiral pinhole aperture as shown in Figure 2(a), respectively. Figure 2(d) presents the corresponding TC spectrum, illustrating the power spectrum of TC modes. The coherence vortex spectra can be tuned to a specific TC mode by adding identical copies of the spiral pinhole plates uniformly distributed along the azimuth as shown in Figure 2(e) and (i). Figure 2(e) shows three spiral pinhole structures evenly allocated along the azimuthal direction. Figure 2(f) and (g) show the corresponding amplitude and phase of spatial coherence function, respectively, and Figure 2(h) is the associated coherence vortex spectra with specific TC mode with TC *l* = −3. Similarly, Figure 2(i) shows five spiral pinhole structures uniformly placed along the azimuthal direction. Figure 2(j) and (k) show the corresponding amplitude and phase of spatial coherence function, respectively, and Figure 2(l) is the associated coherence vortex spectra with specific TC mode with TC *l* = −5. Here, an *m*-order rotationally symmetric structure can only select TC modes that are multiples of *m*, while discarding all other modes, and such features have been demonstrated for the fully coherent beam in Ref. [60], [64]. For this reason, particular TC values *l* = −3 and −5 are visible in the spectra displayed in Figure 2(h) and (l), respectively. If we consider the anti-clockwise rotation of spiral masks instead of clockwise rotation along the azimuth, it finally results in TCs with positive values.

### 2.2 Coherence vortex with ±*l* modes

We also create the superposition of two vortex beams with opposite TC: ± *l*, *i.e.*, photonic gears in coherence function. To create a far-field coherence vortex spectrum with this feature, we have selected $I\left(v\right)$ in such a way that two spiral pinhole masks with opposite TC values are combined. If the total number of pinholes azimuthally placed in clockwise and anticlockwise order are *N* and *M*, respectively. The radius $v\left(n,m\right)$ and azimuthal angle $\theta \left(n,m\right)$ of *n*th pinhole in the *m*th spiral are

(13) $$v\left(n,m\right)=\pm {\left(\frac{lz\lambda \theta \left(n,m\right)}{\pi }+r_{0}^{2}\right)}^{\frac{1}{2}},$$

and

(14) $$\theta \left(n,m\right)=\pm \left(\frac{2\pi n}{N}+\frac{2\pi m}{M}\right).$$

**Figure 3:**
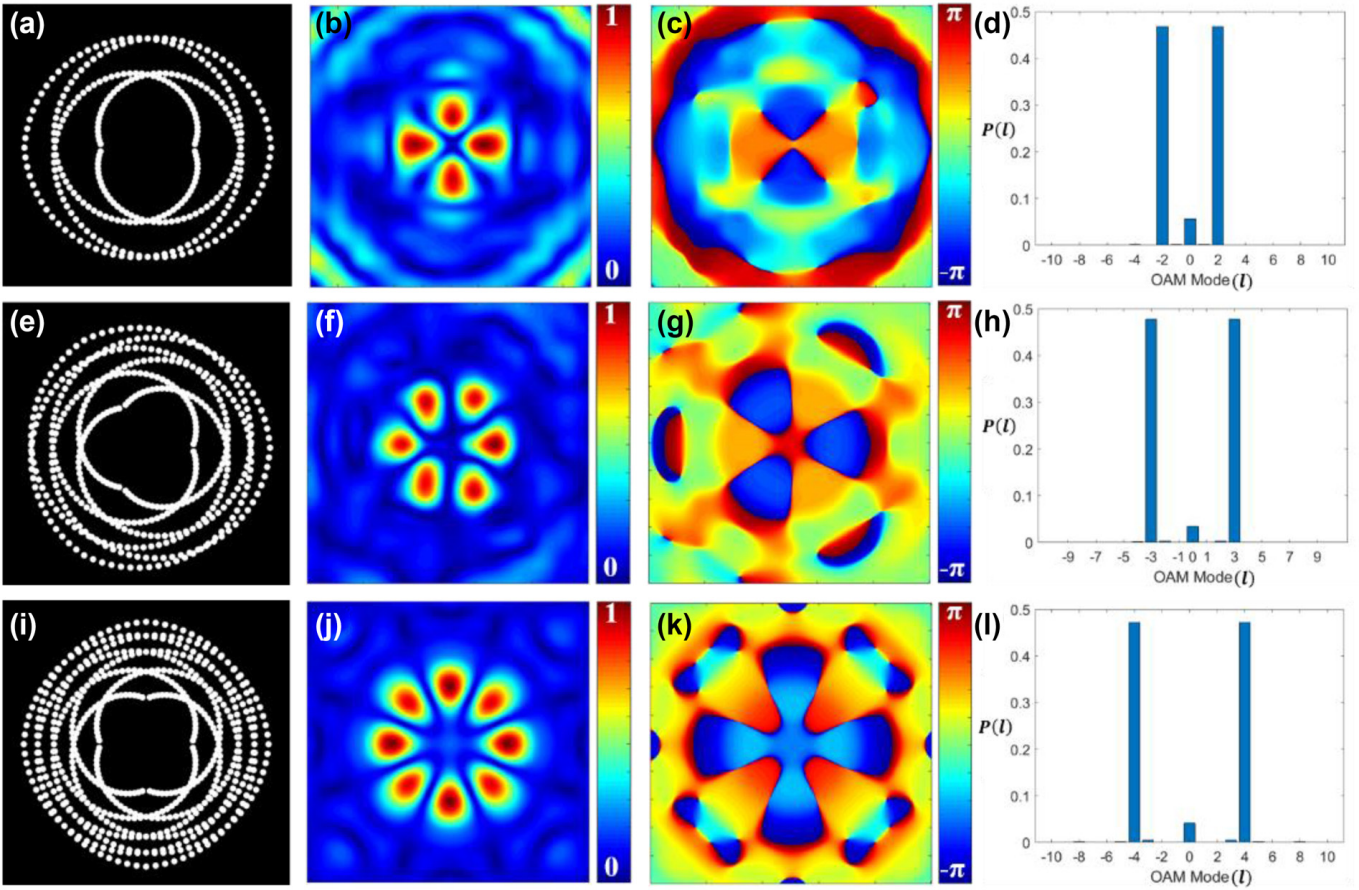
Schematics of the pinhole gears. Binary pinhole masks for OAM modes with (a) *l* = ±2, (e) *l* = ±3, and (i) *l* = ±4; (b), (f), and (j) the corresponding simulated absolute values of complex spatial coherence function; (c), (g), and (k) the corresponding simulated phase values of complex spatial coherence function; (d), (h), and (l) the corresponding TC mode power spectrum.

Following the simulation process described previously, we modeled the propagation of an incoherent beam from the pinhole gears, and the complex coherence results were evaluated at different distances from the aperture plane. Figure 3(a), (e), and (i) show the binary pinhole masks produced through simulation which generates the pinhole gears with superposition of modes *l* = ±2, *l* = ±3, and *l* = ±4, respectively. Figure 3(b), (f), and (j) represent the absolute part of the complex coherence function. The superposition of two vortex beams with opposite TCs ± *l* is known to produce an intensity pattern with 2*l* petal-like features in it [21]. The spatial coherence function also exhibits the same kind of 2*l* petal-like distribution as demonstrated in Figure 3(b), (f), and (j). Figure 3(c), (g), and (k) show the phase distribution of the complex spatial coherence corresponding to Figure 3(a), (e), and (i), respectively. It is demonstrated that the generated coherence vortex mode spectra exhibit highly pure helical modes, as shown in Figure 3(d), (h), and (l), corresponding to Figure 3(a), (e), and (i), respectively.

## 3 Experiment and results

**Figure 4:**
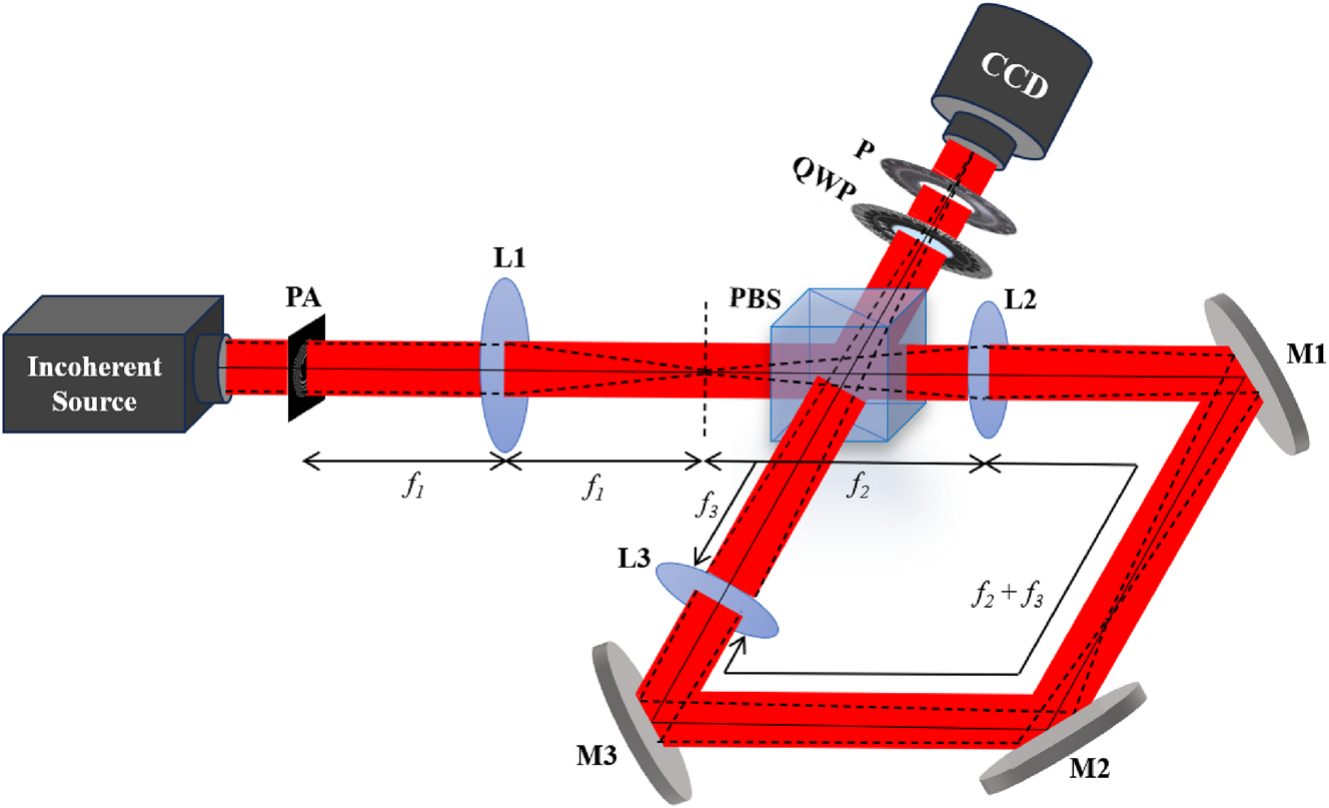
Experimental setup: PA, binary pinhole aperture; L, lens; PBS, polarization beam splitter; M, mirror; QWP, quarter wave plate; P, polarizer; CCD, charge-coupled device.

Following the description of our simulations, experimental tests were carried out to confirm and support our findings. Figure 4 shows our proposed experimental setup. A He–Ne laser of wavelength 632.8 nm illuminates a rotating ground glass disk (RGGD) to generate a spatially incoherent source. Immediately after the RGGD, we placed a binary pinhole aperture (6.5 mm aperture). The pinholes have a diameter of 150 μm. The binary pinhole structures have been realized by lithography, followed by wet etching methods. First, a positive photoresist (S1813 from Micro Resist Technology, Germany) was coated on a 100 nm thick chrome-coated glass substrate. Desired structures were fabricated on this substrate by lithography using a maskless lithography system (SF-100 from Intelligent Micropatterning USA). After development, the substrate was dipped in chrome etchant (Etch-18, from Micro Resist Technology, Germany) for the removal of chrome from exposed parts. Finally, the photoresist was removed and the substrate was cleaned to achieve the desired mask structures. The incoherent source, i.e. transparency is placed at the back focal plane of a bi-convex lens L1 of focal length *f*_1_ = 10 cm, and the back focal plane of this lens is represented by a dotted line in Figure 4. We experimentally measured the complex coherence of the beam at the back focal plane. To measure the complex coherence function arising from the specially designed incoherent source at the far-field as indicated by the black dotted line in Figure 4, we have designed a Sagnac shearing interferometer composed of a polarization beam splitter (PBS), three mirrors M1, M2, and M3, and two lenses L2 and L3 of focal length *f*_2_ = 17.5 cm and *f*_3_ = 20 cm, respectively. The incoming beam is split into *x* and *y* polarized beams by the PBS. The interferometer is configured to ensure that the two counter-propagating beams undergo magnification and demagnification with magnification factors *α* = *f*_3_/*f*_2_ = 1.143 and *α* ^−1^ = *f*_2_/*f*_3_ = 0.875, respectively. Consequently, at the output of the interferometer, we observe two sheared copies of the counter-propagating beams reaching the CCD plane. Thus, at any position in the detector, we have two scaled replicas of the fields with scaling ***r***_1_ = *α* ^−1^ ***r*** and ***r***_2_ = *α* ***r***. Before the detector, a quarter wave plate (QWP) and a polarizer (P) are placed to implement the four-phase shifting technique. The QWP changes the polarization states, converting the *x*-polarized beams to right circular polarization and the *y*-polarized beams to left circular polarization. Later, polarizer will give the required phase shift and help the two orthogonal beams to interfere [65]. Therefore, at the detector plane, the average intensity is determined as

(15) $$\begin{array}{ll} I\left(\theta \right) & \approx I\left(r_{1}\right)+I\left(r_{2}\right)+2\sqrt{I\left(r_{1}\right)}\sqrt{I\left(r_{2}\right)}g\left(\Delta r\right)\\ & \;\times \cos\left[\phi \left(\Delta r\right)+2\theta \right], \end{array}$$

where $I\left(\theta \right)$ is the total average intensity and $I\left(r_{1}\right)$ , and $I\left(r_{2}\right)$ are average intensities at points ***r***_1_ and ***r***_2_. $g\left(\Delta r\right)$ is the fringe visibility and $\phi \left(\Delta r\right)=\phi \left(r_{1}\right)-\phi \left(r_{2}\right)$ is the corresponding phase due to the two interfering beams, and 2*θ* is the constant phase resulting from the polarization rotation. Therefore, a phase shift of 2*θ* is introduced between the two interfering beams by rotating polarizer P by an angle *θ*.

Therefore, four interference patterns $I\left(0\right)$ , $I\left(\frac{\pi }{2}\right)$ , $I\left(\pi \right)$ and $I\left(\frac{3\pi }{2}\right)$ are recorded with phase-shifts 0, π/2, π, and 3π/2, respectively. Using the four-phase shifting algorithm, the fringe visibility and corresponding phase will be determined from these interference patterns, as described by

(16) $$g\left(\Delta r\right)\propto \frac{\sqrt{{\left[I\left(0\right)-I\left(\pi \right)\right]}^{2}+{\left[I\left(\frac{\pi }{2}\right)-I\left(\frac{3\pi }{2}\right)\right]}^{2}}}{I\left(0\right)+I\left(\frac{\pi }{2}\right)+I\left(\pi \right)+I\left(\frac{3\pi }{2}\right)},$$

and

(17) $$\phi \left(\Delta r\right)=\tan^{-1}\left[\frac{I\left(\frac{3\pi }{2}\right)-I\left(\frac{\pi }{2}\right)}{I\left(\pi \right)-I\left(0\right)}\right].$$

Using Eqs. (16) and (17) the complex spatial coherence function is reconstructed as

(18) $$W\left(\Delta r\right)=g\left(\Delta r\right)\exp\left[i\phi \left(\Delta r\right)\right].$$

**Figure 5:**
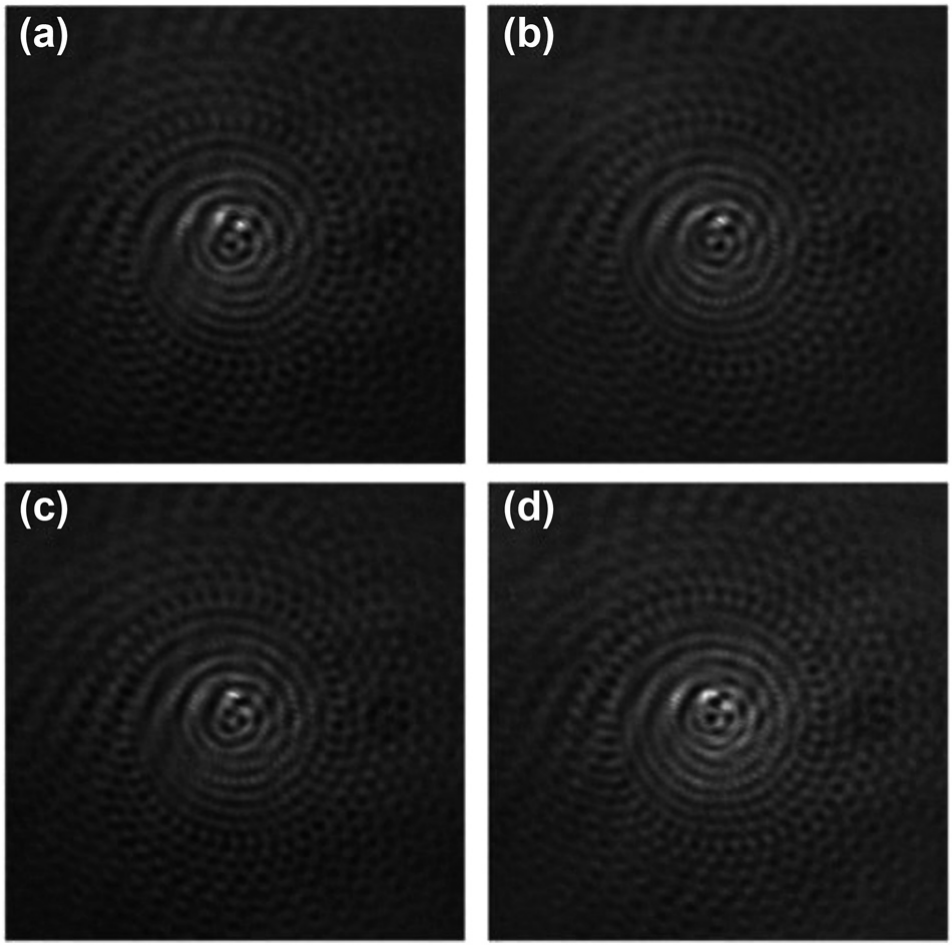
Recorded interference patterns for a single spiral with phase-shifts (a) 0, (b) π/2, (c) π, and (d) 3π/2.

**Figure 6:**
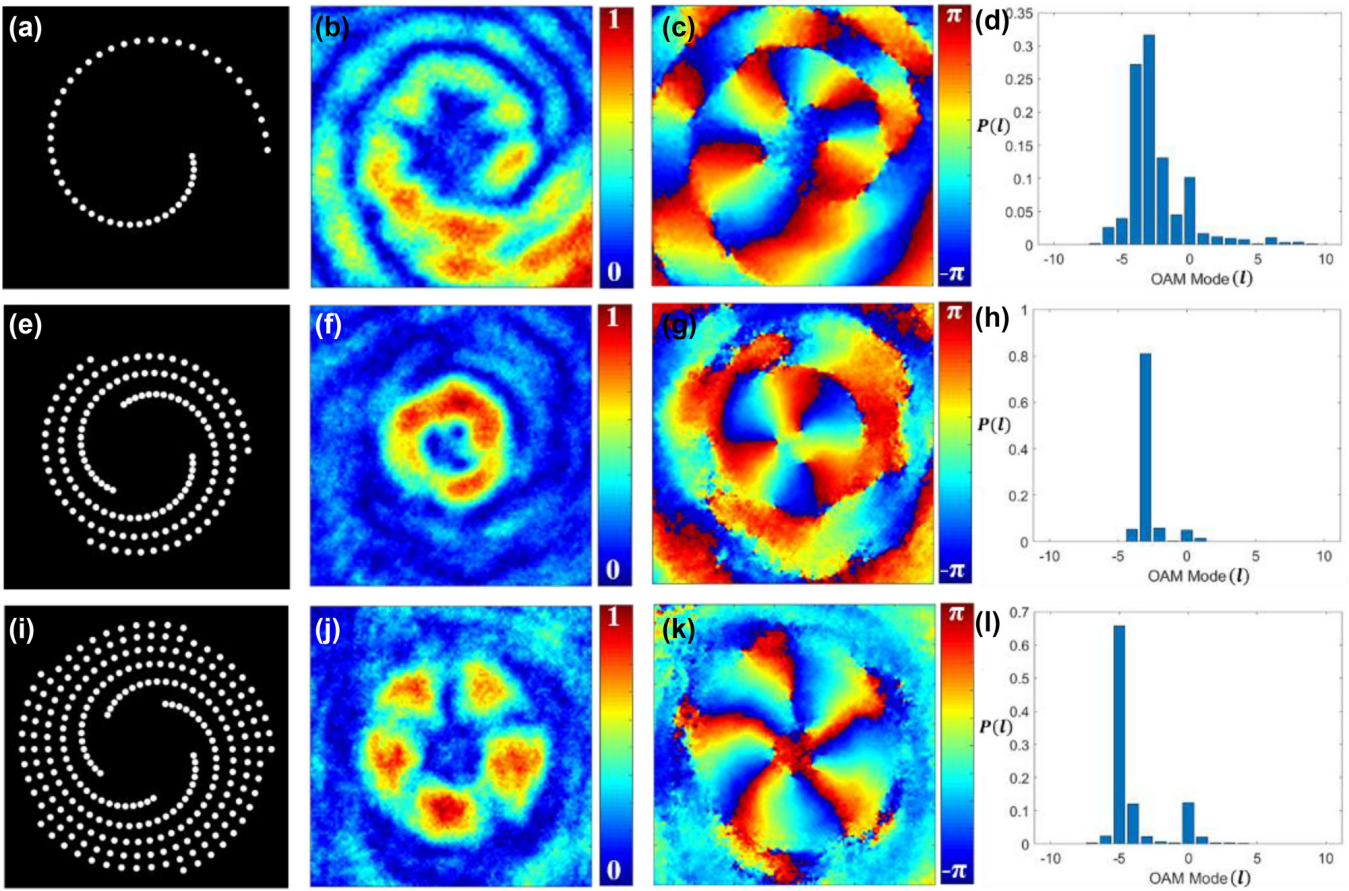
Experimental results. (a) Single spiral, (e) three spirals, and (i) five spirals; (b), (f), and (j) the corresponding absolute values of the complex spatial coherence function; (c), (g), and (k) the corresponding phase values of the complex spatial coherence function; (d), (h), and (l) the corresponding TC mode power spectrum.

Figure 5 represents four experimentally recorded interference patterns $I\left(0\right)$ , $I\left(\frac{\pi }{2}\right)$ , $I\left(\pi \right)$ and $I\left(\frac{3\pi }{2}\right)$ with phase-shifts 0, π/2, π, and 3π/2, respectively, at the CCD plane for single spiral pinhole aperture. These interference patterns were processed and converted into grayscale image using MATLAB for visual enhancement. Later, the complex spatial coherence function is digitally reconstructed using Eqs. (16)–(18). Similarly, the interference patterns corresponding to different spiral pinhole apertures placed after RGGD are recorded, and the results for reconstructed complex spatial coherence function and corresponding coherence vortex spectrum are presented in Figure 6.

Figure 6 represents our experimental results corresponding to simulation results presented in Figure 2. Figure 6(a), (e), and (i) display the binary spiral pinhole apertures with a single spiral, three spirals, and five spirals, respectively. Figure 6(b), (f), and (j) represent the experimental results for the corresponding absolute value of complex spatial coherence and Figure 6(c), (g), and (k) are the corresponding phase values of complex spatial coherence. Figure 6(d), (h), and (l) present the corresponding coherence vortex spectrum, illustrating the power weight distribution of helical modes. Therefore, the experimental results match well with the simulation results presented in Figure 2.

**Figure 7:**
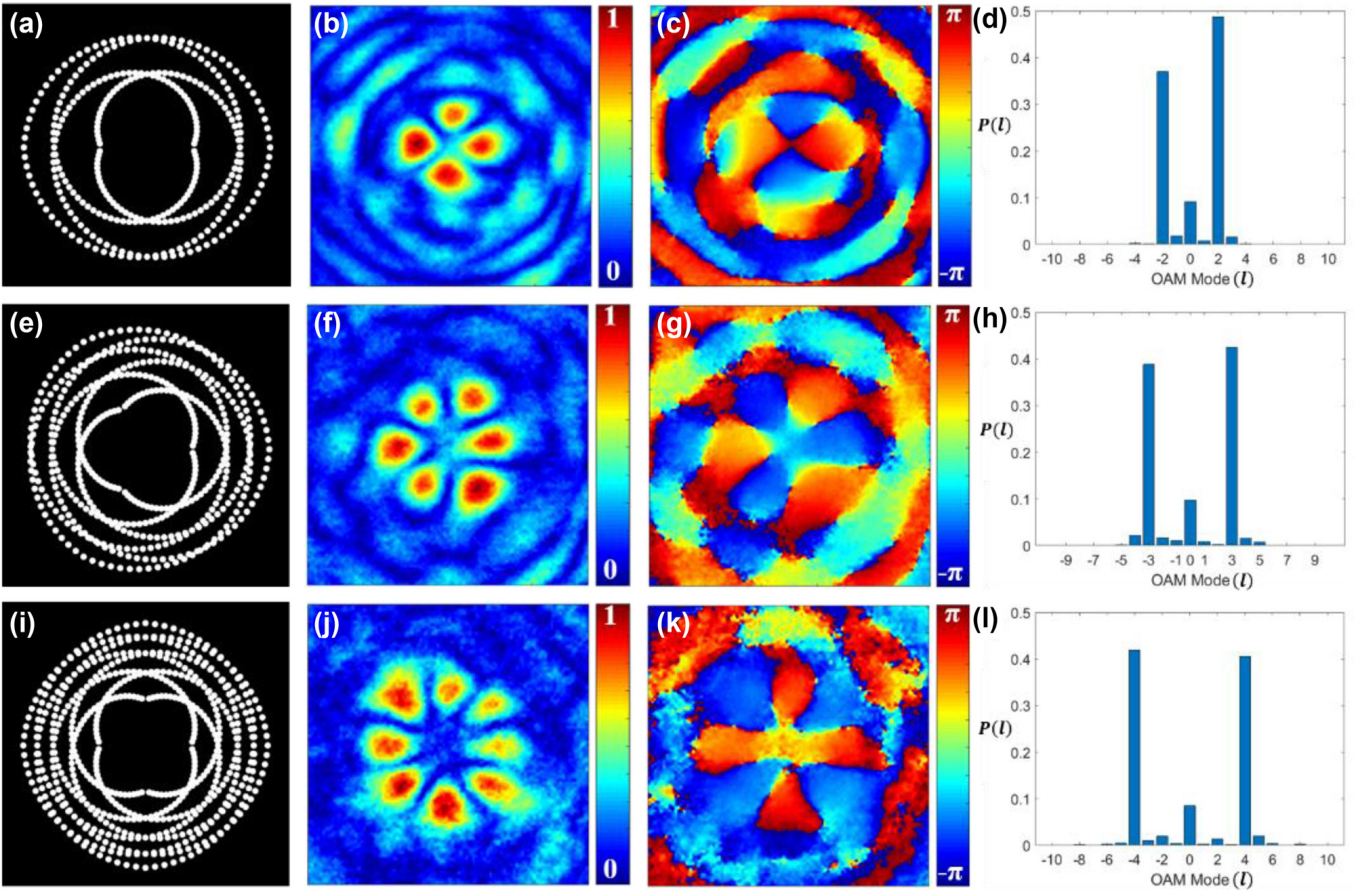
Experimental result for pinhole gears. Binary pinhole masks for TC modes with (a) *l* = ±2, (e) *l* = ±3, and (i) *l* = ±4; (b), (f), and (j) the corresponding absolute values of complex spatial coherence function; (c), (g), and (k) the corresponding phase values of complex spatial coherence function; (d), (h), and (l) the corresponding TC mode power spectrum.

Figure 7 represents our experimental results corresponding to simulation results presented in Figure 3 for pinhole gears. Figure 7(a), (e), and (i) show the binary pinhole masks with superposition of modes *l* = ±2, *l* = ±3, and *l* = ±4, respectively. Figure 7(b), (f), and (j) represent the experimental results for the corresponding absolute value of complex spatial coherence and Figure 7(c), (g), and (k) are the corresponding phase values of complex spatial coherence. Figure 7(d), (h), and (l) present the corresponding coherence vortex spectrum, illustrating the power spectrum of helical modes. The experimental results align closely with the simulation results shown in Figure 3.

## 4 Conclusions

In conclusion, we have presented a novel technique to generate the coherence vortex spectrum of pure and multiple helical modes with spatially designed binary spiral pinhole plates. A thorough theoretical foundation is established, and an experimental technique is proposed and validated through simulations. For the experiment, a Sagnac shearing interferometer is designed to record the complex two-point spatial coherence function, thereby revealing the generation of coherence vortices within it. The absolute part of the complex coherence function displays a doughnut structure whereas the phase part reveals a generic helical phase profile. The coherence vortex spectrum of TC modes is also analyzed using the orthogonal projection method. The spiral pinhole plate helps to tailor the coherence vortex spectrum which can find potential applications in a variety of OAM-based systems. The generation of photonics gears is also presented using the same experimental setup. The present idea is not limited to the optical regime, but can further be explored for matter waves.
